# Characteristics of lower airway parameters in an adult Asian population related to endotracheal tube design: a cadaveric study

**DOI:** 10.1038/s41598-024-56504-5

**Published:** 2024-03-13

**Authors:** Chairat Turbpaiboon, Adisak Kasemassawachanont, Jirawat Wankijcharoen, Kittipott Thusneyapan, Pramuk Khamman, Karnkawin Patharateeranart, Ramida Amornsitthiwat, Terasut Numwong, Nophanan Chaikittisilpa, Taniga Kiatchai

**Affiliations:** 1 Department of Anatomy, Faculty of Medicine Siriraj Hospital, Mahidol University, Bangkok, Thailand; 2 Department of Anesthesiology, Faculty of Medicine Siriraj Hospital, Mahidol University, 2 Wanglang Road, Bangkok Noi, Bangkok, 10700 Thailand; 3 Department of Radiology, Faculty of Medicine Siriraj Hospital, Mahidol University, Bangkok, Thailand

**Keywords:** Anatomy, Adverse effects

## Abstract

The risk of endotracheal tube (ETT) placement includes endobronchial intubation and subglottic injury. This study aimed to describe the lengths of lower airway parameters related to cuff location and vocal cord markings in different adult-sized ETTs. Eighty cadavers were examined for the lengths of the lower airway, including their correlations and linear regressions with height. Thirty adult-sized ETTs from seven different brands were examined for Mark-Cuff and Mark-Tip distances. The depth of ETT placement was simulated for each brand using vocal cord marking. The mean (standard deviation) lengths from the subglottis, trachea, vocal cord to mid- trachea, and vocal cord to carina were 24.2 (3.5), 97.9 (8.6), 73.2 (5.3), and 122.1 (9.0) mm, respectively. Airway lengths were estimated as: (1) subglottis (mm) = 0.173 * (height in cm) − 3.547; (2) vocal cord to mid-trachea (mm) = 0.28 * (height in cm) + 28.391. There were variations in the Mark-Cuff and Mark-Tip distances among different ETTs. In the simulation, endobronchial intubation ranged between 2.5 and 5% and the cuff in the subglottis ranged between 2.5 and 97.5%. In summary, the lower airway parameters were height-related. ETT placement using vocal cord marking puts the patient at a high risk of cuff placement in the subglottis.

## Introduction

The ideal depth of the endotracheal tube (ETT) should have a tip at mid-trachea to prevent endobronchial intubation and a cuff below the cricoid outlet to avoid subglottic injury. This is particularly challenging during the intraoperative period owing to the lack of routine radiographic confirmation. Variations in the tracheal length have been reported in multiple studies with different racial populations^1^, including Indian^2–4^, Asian^5–8^, and Caucasian^9^. The length of vocal cord-to-carina (VC-Carina) in males were larger than females, and also correlated with height^5–7^; however, the current practice of ETT selection takes only sex and airway diameter into account. The effect of race and sex with the same height on airway parameters remains unknown.

Moreover, changes in tracheal length within the same individual have also been reported as a result of dynamic clinical factors^10^. Neck flexion, Trendelenburg position, and pneumoperitoneum result in tracheal shortening, thereby increasing the risk of endobronchial intubation^11–16^. On the other hand, neck extension results in tracheal elongation, leading to potential subglottic injury and accidental extubation^11–13,17^. The mean distance between the tip of the ETT to the carina (Tip-Carina) decreases by 0.6–1.9 cm during head and neck flexion and increases by 0.6–2.7 cm during head and neck extension^10,13^. The vocal cord marking (VCmarking) was introduced to ensure the appropriate depth of endotracheal intubation^18^. However, despite guidance from VCmarking, inappropriate placement still occurs. Up to 30–50% of the patients who were intubated were reported to be at risk of endobronchial intubation as Tip-Carina distance < 3 cm^2,4^. Kang et al. also reported that 6.7% of the patients had cuff displacement beyond the cricoid cartilage after head and neck extension for thyroidectomy^17^.

We hypothesized that an ETT with VCmarking designed to be used globally might not be appropriate for the Asian population. The advancement of this cadaveric study was the clearly demarcated laryngeal and tracheal anatomy that were difficult to measure in a living intubated patient, therefore, we would be able to report the incidence of both the cuff in the subglottis and endobronchial intubation. We aimed to (1) describe the lengths of lower airway parameters related to endotracheal intubation and their correlation with height, and (2) describe the locations of cuff and VCmarking in different adult-sized ETTs and investigate the potential problems related to these variations. We also proposed a method for calculating the appropriate distance from the VCmarking to the upper edge of the cuff and the tip on the ETT.

## Methods

This descriptive cadaveric study was conducted in the Department of Anatomy, Faculty of Medicine Siriraj Hospital, Mahidol University, Bangkok, Thailand. The research protocol complied with a research exemption from the Siriraj Institutional Review Board, SIRB Protocol No. 341/2565 (exempt). The requirement for written informed consent was waived by the institutional review board. This manuscript adheres to the Strengthening the Reporting of Observational Studies in Epidemiology (STROBE) guidelines. All methods were carried out in accordance with relevant guidelines and regulations.

### Cadaveric data collection

**Figure 1:**
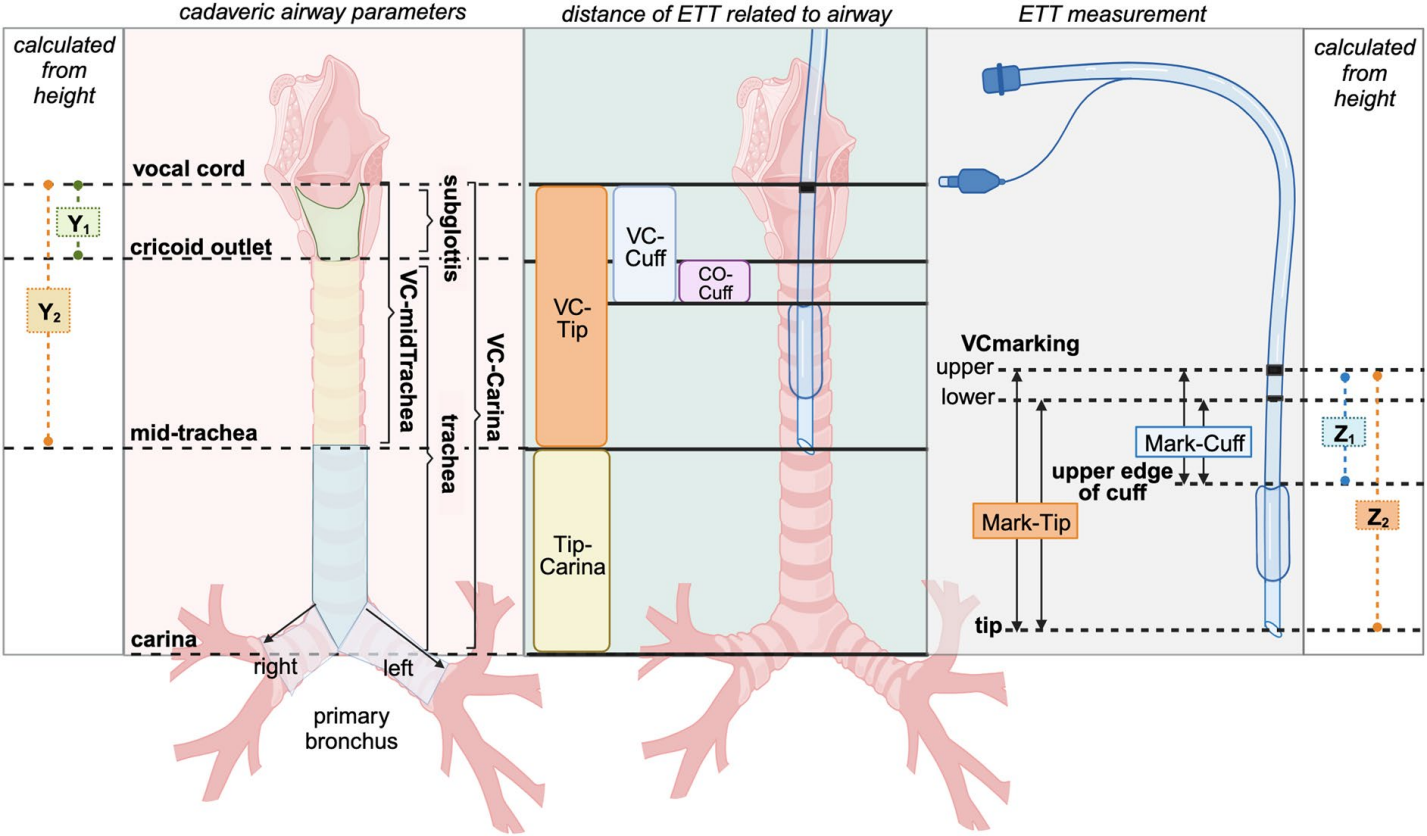
Cadaveric airway parameters, endotracheal tube (ETT) measurements and distances of the cuff and tip related to the vocal cord (VC), cricoid outlet (CO), and carina. The height-estimated airway parameters are presented as Y_1_ (estimated subglottic length) and Y_2_ (VC-mid-tracheal length). The height-estimated appropriate ETT distances are presented as Z_1_ (expected Mark-Cuff) and Z_2_ (expected Mark-Tip). (Created with BioRender.com).

Eighty cervical and thoracic specimens from formalin-embalmed cadavers (40 males, 40 females) donated to the Department of Anatomy, Faculty of Medicine, Siriraj Hospital, Mahidol University, Thailand, for academic and research purposes were included in this study. Cadavers with physical evidence of midline neck and chest injuries, deformities, or previous surgical intervention were excluded. Demographic information, including age, sex, and height, was collected. The height was retrieved from the record before death. The specimens were measured by one of the four investigators (CT, AK, JW, and KT) using an electronic sliding caliper with confirmation of outlying data. The lower airway parameters of interest were the lengths of subglottis, and trachea. The subglottis length was defined as the vertical distance from the medial edge of the vocal cord (VC) to the cricoid outlet. The trachea length was measured as the vertical distance from the cricoid outlet to the carina. If possible, the longitudinal length of both primary bronchi was measured from the upper border of the bronchus, starting from the tracheal bifurcation to the lower border at the branching level of the secondary bronchi. The lengths from the medial edge of the VC to the mid-trachea position and the carina (VC-midTrachea and VC-Carina, respectively) of each cadaver were inferred from the measured data. All measured and inferred parameters are illustrated in Fig. 1.

### Endotracheal tube data collection

Seven different ETT brands with inner diameters (ID) from 6.5 to 8.5 mm were ordered from their local distributors between March and August 2022. The available brands included A: Portex (Ref 100/199/065-085; Smiths Medical International Ltd., Hythe, Kent, UK); B: Ruschelit (Ref 112482; Teleflex Medical Sdn. Bhd., Kamunting, Perak, Malaysia); C: Shiley™ TaperGuard (Ref 18765–18785; Covidien LLC, Mansfield, MA, USA); D: Curity® (Ref 9465E, 9570E, 9475E-9485E; Covidien LLC, Mansfield, MA, USA); E: Unomedical™ (Ref UM61110065-85; Well Lead Medical Co., Ltd., Guangzhou, P.R.China); F: Fornia™ (Ref QG-P2-7.0 to 8.0; Royal Fornia Medical Equipment, Co., Ltd., Guangdong, P.R. China); and G: MICROCUFF® (Ref 35214-5; Avanos Medical Inc., Alpharetta, GA, USA). Each ETT was ordered for two samples. Both samples were measured by two independent investigators (JW and PK) using an electronic sliding caliper (Digimatic Caliper; Model No. CD-6 ASX, Mitutoyo Corporation, Kawasaki-shi, Kanagawa, Japan), resulting in four measurements for each ETT. The inner and outer diameters were determined according to the manufacturer’s descriptions. The distances from the mid-point of the VCmarking to the upper edge of the cuff and the tip of the ETT (Mark-Cuff and Mark-Tip, respectively) were measured (Fig. 1). If the VCmarking was wide, the distance was calculated using the average value of the upper and lower edges of the VCmarking.

### Simulation of different ETT locations in cadaveric samples

We combined the cadaveric and ETT data to simulate ETT placement in three scenarios. First, the VCmarking was positioned at the vocal cord level, to investigate whether the VCmarking is suitable to indicate the appropriate tip and cuff position in the trachea. Distances of ETT related to the airway parameters were calculated including: VC to the tip (VC-Tip), VC to the upper edge of the cuff (VC-Cuff), cricoid outlet to the upper edge of the cuff (CO-Cuff), and the tip to carina (Tip-Carina) (Fig. 1). Second, the tip was positioned at mid-trachea, we investigated whether the upper edge of the cuff was located at the level of the subglottis. Third, in case that the upper edge of the cuff was positioned just below the cricoid outlet using height-estimated regression, we explored if the tip was located too close to the carina (< 2 cm). ETT with an ID of 7.5 and 8.0 mm were tested in males, while the ones with 7.0 and 7.5 mm were tested in females. According to the previous studies showing that the ETT may displace during movement or surgical positioning^10–17^, we further estimated a safe buffer zone of 2 cm for upward or downward ETT displacement. Therefore, the proper position of the upper edge of the cuff was expected to be at least 2 cm below the cricoid outlet (CO-Cuff > 2 cm), and the proper position of the ETT tip was at least 2 cm above the carina (Tip-Carina > 2 cm). The percentage of ETT positions violating the preferred ranges was determined in this study.

### Sample size calculation

In a study conducted within the same ethnicity, Thai anesthetized patients, Techanivate et al.^5^ reported a mean VC-Carina length of 12.33 cm (standard deviation 1.53 cm) with a correlation with height (Pearson’s correlation, *r* = 0.557). We calculated the sample size based on an alpha error probability at 0.05, the power (1 – beta error probability) of 0.80, the two-tailed model, and the moderate effect size at 0.33 (approximately 60% of the Pearson’s correlation coefficient reported by Techanivate et al.^5^), using the G*power program version 3.1.9.7^19^. The required sample size was 69.

### Statistical analysis

Statistical analyses were conducted using SPSS program version 18.0.0 (PASW Statistics 18, Chicago, IL, USA). Descriptive statistics were presented as the mean, standard deviation (SD), coefficient of variance (CV), 95% confidence interval (CI), minimum, and maximum. Each parameter was analyzed in both total and sex-categorized cadavers. The statistical significance of the difference in means between males and females was verified using an unpaired t-test. Height and airway parameters were tested for normal distribution using the Kolmogorov–Smirnov and Shapiro–Wilk tests for the numbers of studied specimens greater and lower than 50, respectively. Pearson’s linear correlation analysis was conducted for all combinations of the height and lower airway parameters. The interpretation of Pearson’s correlation coefficient (*r*) is defined as negligible (0.00–0.10), weak (0.10–0.39), moderate (0.40–0.69), strong (0.70–0.89), and very strong (0.90–1.00)^20^. The statistically significant correlation results were subject to the linear regression analysis to find the regressed equations of those paired parameters. Endobronchial intubation and the cuff in the subglottis were reported as numbers (%) and 95% CI using the Wilson Score method. Missing data was excluded from the analysis.

## Results

### Cadaveric measurement

Descriptive statistics for demographic and lower airway parameters are presented in Table 1. All laryngeal and tracheal parameters were collected from 80 cadavers. The mean (SD) length of the subglottis, trachea, VC-midTrachea, and VC-Carina were 24.2 (3.5), 97.9 (8.6), 73.2 (5.3), and 122.1 (9.0) mm, respectively. Among the data for all lower airway parameters, the length of the subglottis and VC-midTrachea had the highest and lowest data dispersion (based on the CV), respectively. Notably, a significant difference in means between different sexes (males’ greater than females’) was observed in height and all the length of the lower airway parameters, except for the trachea. These data imply the involvement of the subglottic length in most of the statistically significant parameters. The length of both primary bronchi was provided in Supplemental Digital Content 1.

**Table 1:** Demographic data and lower airway parameters.

Distance	Total				Male				Female				*p*-value
	n	Mean	SD (CV%)	95% CI [min, max]	n	Mean	SD (CV%)	95% CI [min, max]	n	Mean	SD (CV%)	95% CI [min, max]	
Age (year)	80	74.0	12.6 (17.1)	71.2–76.8 [40, 99]	40	74.7	11.5 (15.5)	71.0–78.4 [51, 99]	40	73.3	13.7 (18.7)	68.9–77.7 [40, 93]	0.611
Height (cm)	80	160.0	7.4 (4.6)	158.4–161.7 [145, 175]	40	165.4	5.4 (3.3)	163.7–167.1 [155, 175]	40	154.7	4.9 (3.2)	153.1–156.3 [145, 165]	< 0.001
Subglottis (mm)	80	24.2	3.5 (14.4)	23.4–25.0 [15.7, 32.4]	40	25.8	3.2 (12.4)	24.8–26.9 [18.1, 32.4]	40	22.6	3.0 (13.2)	21.6–23.5 [15.7, 30.2]	< 0.001
Trachea (mm)	80	97.9	8.6 (8.8)	96.0–99.8 [78.2, 122.7]	40	99.0	7.9 (8.0)	96.4–101.5 [78.2, 117.1]	40	96.8	9.2 (9.5)	93.8–99.7 [78.3, 122.7]	0.259
Vocal cord to mid-trachea (mm)	80	73.2	5.3 (7.3)	72.0–74.3 [59.0, 89.5]	40	75.3	5.2 (6.9)	73.7–77.0 [59.0, 89.5]	40	71.0	4.5 (6.4)	69.5–72.4 [59.8, 80.7]	< 0.001
Vocal cord to carina (mm)	80	122.1	9.0 (7.4)	120.1–124.1 [98.1, 148.1]	40	124.8	8.7 (6.9)	122.0–127.6 [98.1, 148.1]	40	119.4	8.6 (7.2)	116.6–122.1 [102.1, 142.0]	0.006

Data were separated into three groups based on sex: total, male, and female. Statistics included mean, SD (standard deviation), CV (coefficient of variance), 95% CI (confidence interval), min (minimum), max (maximum), and *p*-value showing statistically significant differences in means between males and females.

There was no significant deviation from the normal distribution of the height or lower airway parameters. We identified a weak correlation between height and the following airway parameters: the subglottis (*r* = 0.369, *p* = 0.001), VC-midTrachea (*r* = 0.390, *p* < 0.001), and VC-Carina (*r* = 0.318, *p* = 0.004). (Table 2) Linear regression analysis was only conducted for paired parameters with significant correlation to determine the correlation equation for airway parameter estimation (Table 2). The length of estimated subglottis (Y_1_ = 0.173*(height in cm) − 3.547) and estimated VC-midTrachea (Y_2_ = 0.28*(height in cm) + 28.391) were then be applied to determine the expected ETT position based on height.

**Table 2:**
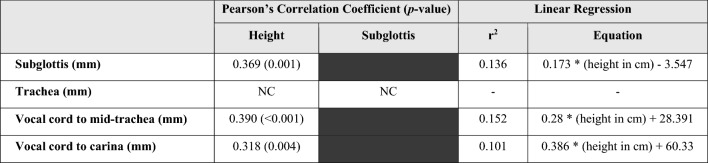
Correlation between parameters including height and airway parameters with their linear regression Pearson’s correlation included the analyses between height and all airway parameters and between some unrelated pairs of airway parameters with their correlation coefficients (*r*) and statistical significance level (*p*-value) provided. Linear regression results (*r*^2^ and regressed equation) were shown only in pairs with statistical significance (*p*-value < 0.05). (NC, no linear correlation).

### Endotracheal tube measurement

**Figure 2:**
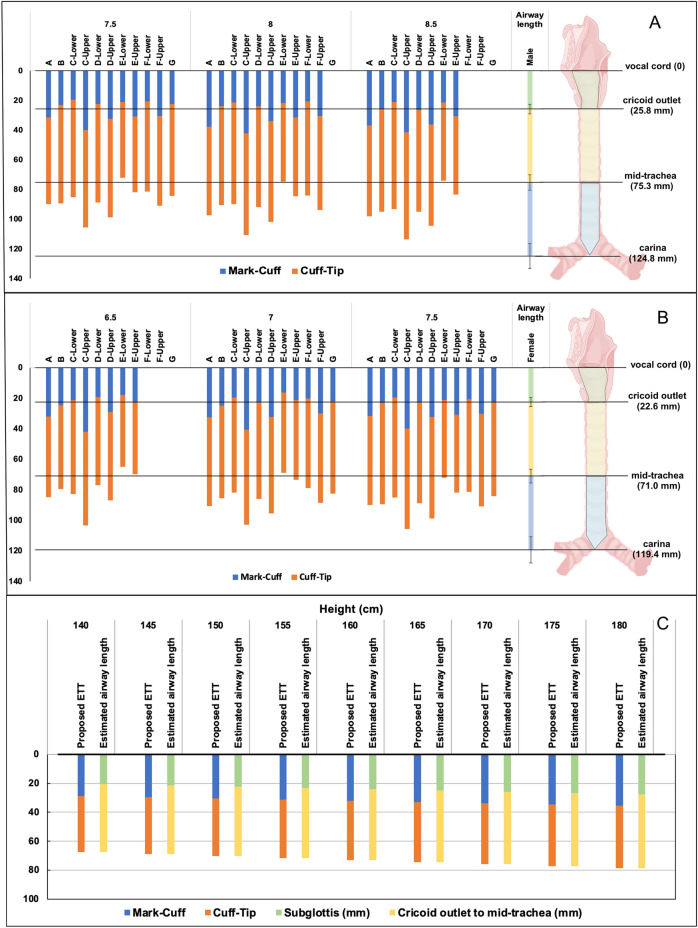
VCmarking to upper edge of cuff (Mark-Cuff) distances (blue) and upper edge of cuff to tip (Cuff-Tip) distances (orange) of different endotracheal tubes in male (**A**), female (**B**), and proposed height-estimated distances (**C**). Mean (standard deviations) of lengths of subglottis (green), cricoid outlet to mid-trachea (yellow), and mid-trachea to carina (light blue) for male and female are presented on the right side of each chart. (Created with BioRender.com).

Thirty ETTs (sizes 6.5–8.5) were available in the market during the study period and included in the study. The Mark-Cuff and Mark-Tip distances varied between brands with the same ID. The ranges of Mark-Cuff distance were 18.0–42.0 mm (ID 6.5), 16.4–40.7 mm (ID 7.0), 19.4–40.0 mm (ID 7.5), 20.6–42.1 mm (ID 8.0), and 21.1–41.6 mm (ID 8.5). The ranges for Mark-Tip distance were 64.9–103.4 mm (ID 6.5), 68.7–102.7 mm (ID 7.0), 72.1–105.8 mm (ID 7.5), 74.8–111.1 mm (ID 8.0), and 73.7–114.0 mm (ID 8.5). To illustrate the ETT ranges in comparison with the mean airway anatomy, the proportional lengths in the bar chart were plotted in Fig. 2A for males and 2 B for females. The details of the other ETT parameters were available in Supplemental Digital Content 2.

### Simulation of ETT placement in cadaveric samples

**Figure 3:**
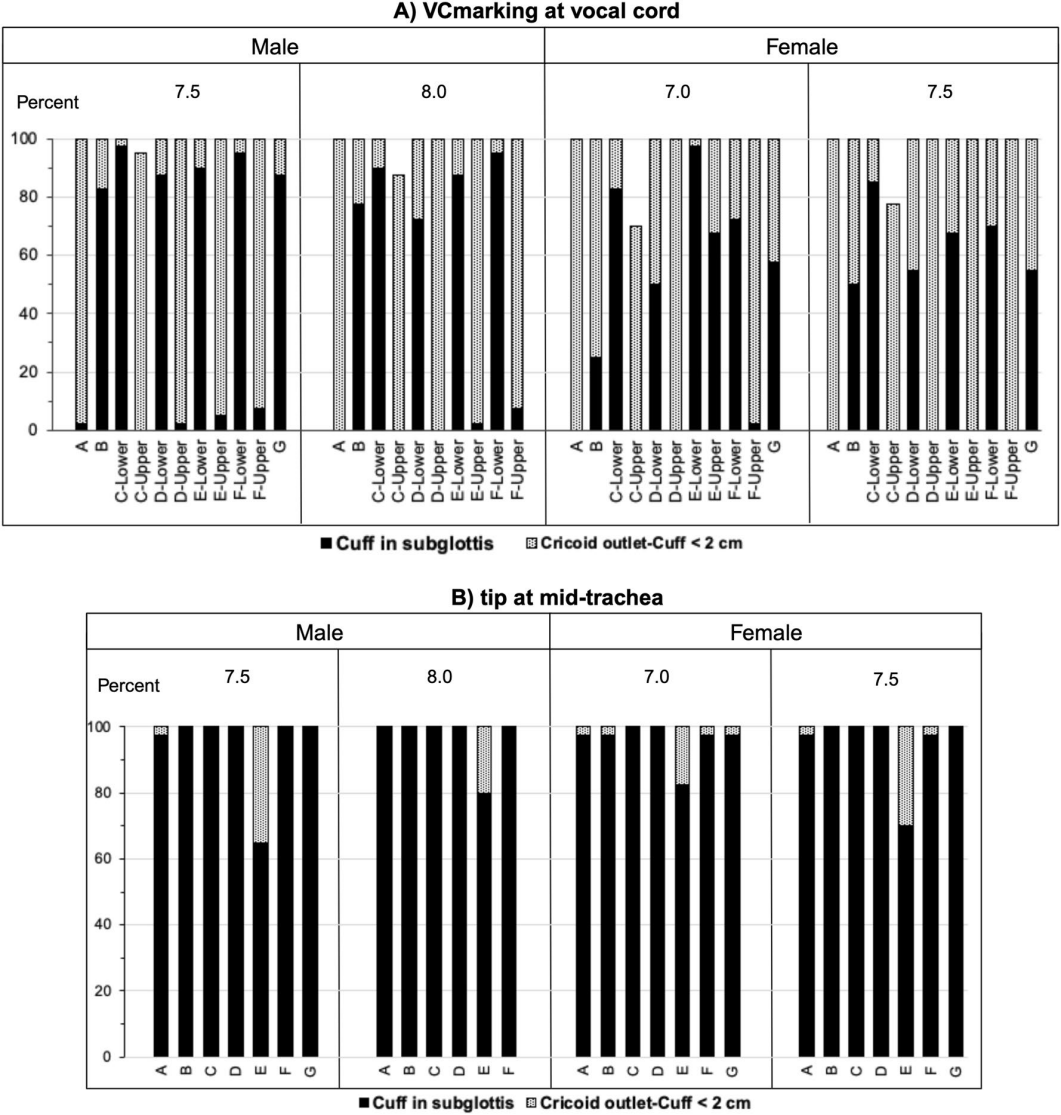
Simulated percentage of potential cuff in subglottis in cadaveric samples for different endotracheal tubes if (**A**) the manufacturer-guided vocal cord markings (VCmarking) were at the vocal cord level or (**B**) the tips were located at mid-trachea level. (Created with BioRender.com).

**Figure 4:**
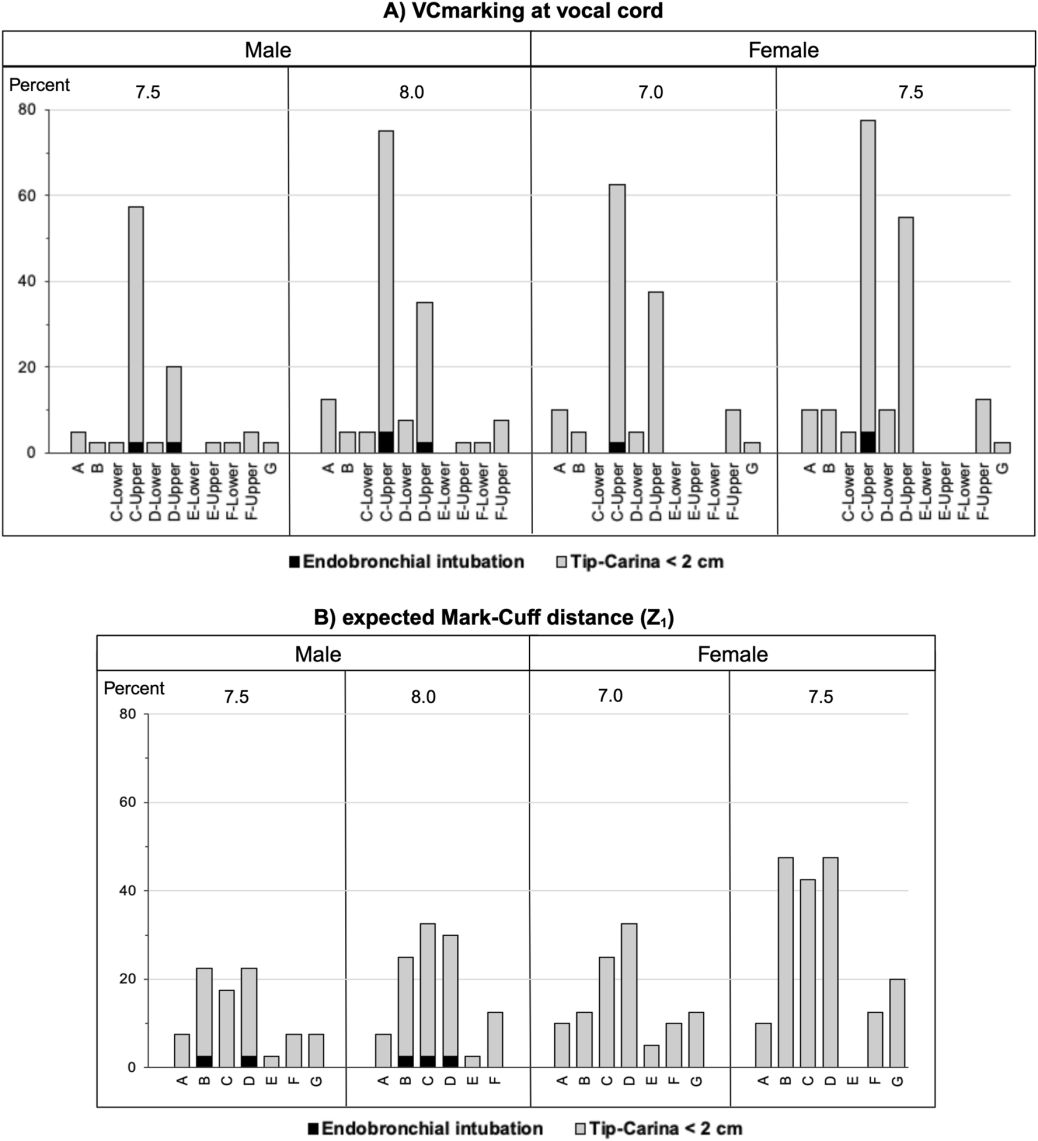
Simulated percentage of potential endobronchial intubation in cadaveric samples for different endotracheal tubes if (**A**) the manufacturer-guided vocal cord markings (VCmarking) were at the vocal cord level or (**B**) using the expected Mark-Cuff (Z_1_) distance. (Created with BioRender.com).

In the first scenario, the percentage of cuff in subglottis and endobronchial intubation for each brand if the VCmarkings were placed at the vocal cord level were demonstrated in Fig. 3A and 4A. There were a total of 43 ETT simulations in both sexes (21 male and 22 female simulations). Each simulation was performed in 40 cadavers. The percentage of cuff in subglottis ranged from 2.5 to 97.5% in 17 out of 21 male simulations and in 14 out of 22 female simulations (Fig. 3A). There was a 2.5–5% occurrence of endobronchial intubation occurred in 4 out of 21 male simulations and in 2 out of 22 female simulations (Fig. 4A). The occurrence of CO-Cuff < 2 cm and Tip-Carina < 2 cm were also illustrated.

In the second scenario, the cuff position of the available ETTs was explored in the simulated ETT placement with the tip located at mid-trachea. In this case, cuff placement in the subglottis was reported to be as high as 65–100% in all 13 simulations in males and 70–100% in all 14 simulations in females (Fig. 3B).

### Simulation of ETT placement using height-estimated parameters

The height-estimated airway parameters of all 80 cadavers were internally validated. For the estimated VC-midTrachea length (Y_2_), the ETT tip in 79 (98.75%) specimens was located within the middle third of the trachea, whereas in 73 (91.25%) specimens, it was located within two middle quarters of the middle third of the trachea. Only one specimen (1.25%) showed a height-estimated mid-tracheal point 1.2 mm below the middle third of the trachea. For the estimated subglottic length (Y_1_), the deviation range of the estimated length from the actual length was within 5 mm for 90% of the studied specimens. However, 44 (55%) specimens had an estimated subglottic length shorter than the actual length, and two specimens in this group had approximately 7 mm (7.422 and 7.787 mm) deviation which was the estimation with the least precision. Therefore, we suggested that the expected Mark-Cuff distance (Z_1_) should be at least 8 mm larger than the estimated subglottis (Z_1_ > estimated subglottis (Y_1_) + 8).

Based on the regression equations of the estimated length of airway parameters in Table 2, the derivation sequence to obtain the equations for the proper ETT position is demonstrated below.

$$Estimated\;subglottis\;(Y_{1} ) = 0.173*(height\;in\;cm) - 3.547\quad \# 1\;(from\;linear\;regression)$$

$$Estimated\;VC{\text -}midTrachea \, ( {Y_{2} } ) = 0.28*( {height\;in\;cm} ) + 28.391\quad \# 2\;( {from\;linear\;regression} )$$

$$Delta\;( {Expected\;Mark{\text -}Tip\;( {Z_{2} } )\;and\;Estimated\;VC{\text -}midTrachea\;( {Y_{2} } )} ):\;minimum\;\;\;\# 3\;( {condition \, 1} )$$

$$Expected\;Mark{\text -}Cuff\;( {Z_{1} } ) > Estimated\;Subglottis\;( {Y_{1} } ) + 8\quad \# 4\;( {condition\;2} )$$

$$Expected\;Mark{\text -}Cuff\;( {Z_{1} } ) > 0.173*( {height\;in\;cm} ) + 4.453\quad \# 5\;{\text{(from}}\;{{\# 1}}\;{\text{and}}\;{{\# 4)}}$$

According to our data, two distances on the ETT need to be calculated when choosing the right ETT: 1) expected Mark-Cuff (Z_1_) distance, and 2) expected Mark-Tip (Z_2_) distance. The expected location of the VCmarking from Z_1_ can be derived from equation #5, The expected location of the VCmarking from Z_2_ can be set from the height-estimated VC-midTrachea (Y_2_) equation below:

$$Expected\;Mark{\text -}Tip\;( {Z_{2} } ) = Estimated\;VC{\text -}midTrachea\;( {Y_{2} } )\quad \# 6\;{\text{(from}}\;{{\# 2}}\;{\text{and}}\;{{\# 3)}}$$

After calculating the expected VCmarkings from Z_1_ and Z_2_ on the ETT, the one that is located further from tip will be used to guide the intubation depth to avoid having a cuff in the subglottis. If the expected VCmarking derived from the expected Mark-Tip (Z_2_ from #6) is placed on the ETT at a location further from tip than the expected VCmarking derived from the expected Mark-Cuff (Z_1_ from #5), then the expected VCmarking derived from Z_2_ will be used to guide the intubation depth (Supplemental Digital Content 3A). The ETT will be safely placed with the appropriate tip position and no cuff in the subglottis. Conversely, if the expected VCmarking derived from the expected Mark-Cuff (Z_1_ from #5) is placed on the ETT at a location further from tip than the expected VCmarking derived from the expected Mark-Tip (Z_2_ from #6), the expected VCmarking derived from Z_1_ will be used to guide the intubation depth to avoid the cuff in the subglottis (Supplemental Digital Content 3B). In this case, there is a potential risk of the tip ending in the lower part of the trachea or endobronchial intubation.

We tested the proposed equations using commercially available ETTs for the studied specimens categorized based on sex. None of the ETTs had Z_2_ placed on the ETT further from tip than Z_1_, suggesting that there was no ETT with the appropriate Mark-Cuff and Mark-Tip distance in the same one. Therefore, all ETTs must be placed based on Z_1_ with a potential risk of endobronchial intubation. To simulate ETT position using the height-estimated Z_1_ to avoid the cuff in the subglottis (third scenario), the tip locations for each brand was reported in Fig. 4B. There was a 2.5% occurrence of endobronchial intubation in 5 out of the 13 simulations in males, and there was no endobronchial intubation in 14 simulations in females.

To illustrate this, the intubation depth for a 160-cm height patient is calculated from the expected Mark-Cuff (Z_1_) distance of (0.173 * 160) + 4.453 = 32.1 mm, while the Tip-Carina distance could be < 2 cm with this depth (Fig. 4B). However, this calculation was expected to place the cuff just below the cricoid outlet in all patients. To obtain a 2-cm safety margin for head extension, the expected mark-cuff (Z_1_) distance must be increased to 52.1 mm, which put the patients at a higher risk for endobronchial intubation. In contrast, if chest radiography indicated that Tip-Carina distance < 2 cm, the critical care physician would withdraw the ETT upward to keep the ETT tip at mid-trachea. Because of the high possibility of a cuff in the subglottis in this situation (Fig. 3B), it is impossible to simultaneously place an ETT with a Tip-Carina distance of > 2 cm and a CO-Cuff distance of > 2 cm in the current design of available ETTs. Theoretically, the upper edge of the cuff should be located as close as possible to the ETT tip to avoid subglottic injury. Owing to the high risk of the cuff in the subglottis reported in this study, we proposed an ETT design for the ETT manufacturer using the following height-based formula:

$$Expected\;Mark{\text -}Cuff\;( {Z_{1} } ) > 0.6179*( {Expected\;Mark{\text -}Tip;\;Z_{2} } ) - 13.0886\quad {\# 7}\;{\text{(from}}\;{{\# 5}}\;{\text{and}}\;{{\# 6)}}$$

The proposed ETT characteristics obtained by height-based calculations were compared with the height-estimated airway parameters in Fig. 2C. The ETT parameters in #7 were expected to provide VCmarking with the tip positioned around the mid-tracheal point and no cuff in the subglottis. Additional details of the ETT placement simulations were provided in Supplemental Digital Content 4.

## Discussion

The lengths of lower airway parameters in our study were similar to several ethnic groups (Caucasian, Taiwanese, Chinese, New Zealander, and Thai)^10^. Our mean (SD) of VC-Carina length was 12.2 (0.9) cm compared with 12.1–13.3 cm in other studies^2,5–9^. Compared to the same ethnicity, Thai anesthetized patients, we found a small variation in the mean (SD) of VC-Carina length [12.3 (1.5) cm] that might be attributed to the small variation in height across different age groups^5^. The difference between the means of different sexes was observed in the height and most airway parameters except for the lengths of the trachea. Interestingly, these lengths also demonstrated a significant correlation with height in the combined sex analysis (Table 2). This may explain, at least partially, the significant difference in the means between the sexes. Height was correlated with both sexes and these lower airway parameters, implying that airway parameters may not be directly correlated with sex but indirectly correlated with height. Additionally, our correlation findings indicate the involvement of the subglottic length as a key factor in every airway parameter, showing a significant correlation with height.

We were unable to demonstrate the significant correlation between height and tracheal length; however, it appeared to unveil the significant correlation once we incorporated the subglottic length into the analyzed parameter as the VC-Carina length, consistent with previous studies^5,6^. VC-carina length was also correlated with sternal length^6^ and thyrosternal distance (only when VC-Carina was shorter than 11 cm)^7^. However, the linearly regressed equation of the correlation between the height and the VC-Carina from our study (*VC-Carina in mm* = 0.386*(height in cm) + *60.33*) differed from that in a previous study by Techanivate et al^5^. (*VC-Carina in cm* = 0.114*(height in cm) – 5.88) which may be attributed to the different age groups and measurement methods.

To prove the hypothesis of correlation between height and lengths of airway for clinical implementation in airway length estimation, this study achieved this aim. Linear correlation was first hypothesized in previous studies^5,6^. To exclude other non-linear correlation, only the airway parameters which met the statistical assumptions for linear correlation with the height were subject to the Pearson’s correlation test. The others that did not meet these statistical assumptions for linear correlation may somehow belong to the non-linear correlation which was not focused in this study. Again, the linearly correlated pairs with statistically significant *p*-value were tested for the statistical assumptions required prior to the linear regression analysis. Although the strength of the correlation coefficients was weak in all correlated pairs, the height-estimated airway parameters from the linearly-regressed equations were already validated in the cadavers for their clinical practicality and demonstrated the outcomes applicable for the actual ETT intubation. Nonetheless, plain chest radiography or fiberoptic confirmation in living patients after intubation based on the correlation and estimation of airway proposed in this study is still required to provide more evidences for strengthening its appropriate application.

There were variations in both the Mark-Cuff and Mark-Tip distances in the ETTs, either within the same ID but different brands or within the same brand but different IDs. Mark-Cuff and Mark-tip distance varied between 25.7 and 49.1 mm, which was a high proportion compared to VC-midTracheal length (mean (SD) 73.2 (5.3) mm). ETT variations have been reported in both adult- and pediatric sizes^7,11,21–23^. Owing to these high variations, conventional ETT selection based solely on the tracheal diameter or outer diameter of the ETT may not be sufficient. The location of VCmarkings on the ETT should be considered during ETT selection.

The strength of this study is that it enabled subglottic measurements, which cannot typically be obtained using plain chest radiography or fiberoptic confirmation after intubation. Unlike most published studies that focused on Tip-Carina distance^2,4,7,12,14–16^, we found that the rate of cuff in the glottis ranged from one-fourth to approximately 100% with most of the ETTs (Fig. 3A). This risk was previously reported in anesthetized adults^7^, pediatrics^21,23^, and pediatric cadavers^24^. In some brands, upper and lower VCmarkings were available on the ETT in order to have the vocal cord placed between the two VCmarkings. Both VCmarkings were tested in the simulations. We found high percentage endobronchial intubation including the Tip-Carina distance < 2 cm ranged from 2.5 to 77.5% with the use of manufacturer’s upper VCmarkings (Fig. 4A), implying lower practicability of the upper than the lower VCmarkings in the Asian population. However, the lower VCmarkings still put the patient at higher risk (> 50%) of the cuff in the subglottis (Fig. 3A). We demonstrated that having the vocal cord placed too close to either the upper or lower VCmarkings put the patient at risk of an inappropriate position. The Mark-Cuff and Mark-Tip distances from manufacturers varied and should not serve as an ideal position for all patients. Consequently, we conclude that the cuff locations from most manufacturers were attached too close to the VCmarkings. Therefore, we propose a height-based calculation method to estimate the proper Mark-Cuff and Mark-Tip distances.

As we have demonstrated that no currently available ETT design is suitable for both tip and cuff positions, multiple publications have addressed the call for manufacturers to redesign ETT in both adults and pediatrics^7,21,24^. Ideally, the proximal edge of the cuff should be placed as low as possible to avoid subglottic injury. Characteristics of the tracheal cuff, including shape, pressure, or material, have been widely discussed in the literature^25^; however, its location in relation to the ETT and cricoid outlet has rarely been mentioned. Laryngeal injury has been reported even after a short general anesthetic, and complications varied based on the design of the tube and cuff^26^. Endoscopic examination after extubation in an intensive care unit reported that 73% of patients had at least one laryngeal abnormality (edema, ulceration, granulation, or abnormal VC mobility)^27^. In the current practice with the existing ETT design, we recommend close monitoring of cuff pressure as there was a very high possibility of cuff placement in the subglottis when the tip was placed at the desired location.

This study had some limitations. First, it focused on an Asian geriatric population in a cadaveric setting, limiting generalizability to other demographics. The size of intercartilaginous soft tissue between tracheal rings may be affected by the cadaveric preservation process. ETT placement was simulated by ETT-airway length comparison, not the actual placement of ETT in the cadaveric airway which may influence the study results due to the curvature of ETT. External validation is required for living subjects with actual ETT placement to universalize the finding from this study. Additionally, geriatric conditions such as osteoporosis may affect height accuracy. Further study on other younger age groups and other ethnicities is required to compare either the consistency of the finding or the age-dependent variation with this study. Second, the sample size was small. Sex-specific correlations were not studied separately as we found that height was the direct factor contributing to the estimation of airway parameters. However, further studies with larger sample sizes for each sex may elucidate the sex-specific characteristics. Third, subglottis and trachea diameter investigation was hindered by cadaveric unreliability. ETT size selection was based on sex-based recommendations. Although there were a number of limitation found in this study, the levels of statistical significance in the correlation study between the height and the airway parameters provided strong evidence (0.001 < *p* < 0.01) against the null hypothesis of no correlation. Moreover, the height-estimated airway parameters of all 80 cadavers were internally validated for their practicality and provided the satisfying results as reported in the part of *Simulation of ETT placement using height-estimated parameters*. This would at least confirm the universality of our study in clinical practice.

In conclusion, most lower airway parameters in adults were height-related. There are variations in the ETT design, including the location of the marking and cuff location on the ETT, which rarely fit the entire population. The conventional technique of intubation to place the marking at the vocal cord level using available ETTs puts the patient at a high risk of cuff placement in the subglottis. Therefore, adult ETTs should be redesigned to lengthen the Mark-Cuff distance and slightly shorten the Mark-Tip distance to avoid both cuff in the subglottis and endobronchial intubation.

### Supplementary Information

Supplementary Information.

## Data Availability

The datasets generated during and/or analysed during the current study are available from the corresponding author on reasonable request.

## Abbreviations

CI: Confidence interval
CO-Cuff: Cricoid outlet to the upper edge of cuff
CV: Coefficient of variance
ETT: Endotracheal tube
ID: Inner diameter
Mark-Cuff: Vocal cord marking to the upper edge of cuff
Mark-Tip: Vocal cord marking to the tip of the endotracheal tube
*r*: Pearson’s correlation coefficient
SD: Standard deviation
Tip-Carina: Tip of the endotracheal tube to carina
VC: Vocal cord
VCmarking: Vocal cord marking
VC-Carina: Vocal cord to carina
VC-Cuff: Vocal cord to the upper edge of the cuff
VC-midTrachea: Vocal cord to mid-trachea
VC-Tip: Vocal cord to the tip
Y_1_: Estimated subglottis
Y_2_: Estimated vocal cord to mid-trachea
Z_1_: Expected distance from vocal cord marking to the upper edge of the cuff
Z_2_: Expected distance from vocal cord marking to the tip of the endotracheal tube
