# Author Correction: Characteristics of lower airway parameters in an adult Asian population related to endotracheal tube design: a cadaveric study

**DOI:** 10.1038/s41598-024-71384-5

**Published:** 2024-08-29

**Authors:** Chairat Turbpaiboon, Adisak Kasemassawachanont, Jirawat Wankijcharoen, Kittipott Thusneyapan, Pramuk Khamman, Karnkawin Patharateeranart, Ramida Amornsitthiwat, Terasut Numwong, Nophanan Chaikittisilpa, Taniga Kiatchai

**Affiliations:** 1 Department of Anatomy, Faculty of Medicine Siriraj Hospital, Mahidol University, Bangkok, Thailand; 2 Department of Anesthesiology, Faculty of Medicine Siriraj Hospital, Mahidol University, 2 Wanglang Road, Bangkok Noi, Bangkok, 10700 Thailand; 3 Department of Radiology, Faculty of Medicine Siriraj Hospital, Mahidol University, Bangkok, Thailand

Correction to: *Scientifc Reports* 10.1038/s41598-024-56504-5, published online 13 March 2024

The original version of this Article contained an error, where the reference numbers provided were incorrect.

As a result, in the Methods section, under the sub-heading “Endotracheal tube data collection”,

“The available brands included A: Portex (Ref 100/199/065-085; Smiths Medical International Ltd., Hythe, Kent, UK); B: Ruschelit (Ref 100382; Teleflex Medical Sdn. Bhd., Kamunting, Perak, Malaysia); C: Shiley™ TaperGuard (Ref 18765–18785; Covidien LLC, Mansfield, MA, USA); D: Curity® (Ref 9465E-9485E; Covidien LLC, Mansfield, MA, USA); E: Unomedical™ (Ref UM61110065-85; Well Lead Medical Co., Ltd., Guangzhou, P.R.China); F: Fornia™ (Ref QG-P2-7.0 to 8.0; Royal Fornia Medical Equipment, Co., Ltd., Guangdong, P.R. China); and G: MICROCUFF® (Ref 35214-5; Avanos Medical Inc., Alpharetta, GA, USA).”

now reads:

The available brands included A: Portex (Ref 100/199/065-085; Smiths Medical International Ltd., Hythe, Kent, UK); B: Ruschelit (Ref 112482; Teleflex Medical Sdn. Bhd., Kamunting, Perak, Malaysia); C: Shiley™ TaperGuard (Ref 18765–18785; Covidien LLC, Mansfield, MA, USA); D: Curity® (Ref 9465E, 9570E, 9475E-9485E; Covidien LLC, Mansfield, MA, USA); E: Unomedical™ (Ref UM61110065-85; Well Lead Medical Co., Ltd., Guangzhou, P.R.China); F: Fornia™ (Ref QG-P2-7.0 to 8.0; Royal Fornia Medical Equipment, Co., Ltd., Guangdong, P.R. China); and G: MICROCUFF® (Ref 35214–5; Avanos Medical Inc., Alpharetta, GA, USA).

The original Article has been corrected.

