# Supplementary material for: Characteristics of lower airway parameters in an adult Asian population related to endotracheal tube design: a cadaveric study
Source: Sci Rep. 2024 Mar 13;14:6137. doi: 10.1038/s41598-024-56504-5 (PMC10937627; doi:10.1038/s41598-024-56504-5)
Supplement: Supplementary file 1 — Supplementary Information. [file 41598_2024_56504_MOESM1_ESM.pdf]

Supplemental Digital Content 1: Primary bronchi parameters and their correlation

Supplemental Digital Content 1A: Primary bronchi parameters. Data were separated into three groups based on sex: total, male, and female. Statistics included mean, SD (standard deviation), CV (coefficient of variance), 95% CI (confidence interval), min (minimum), max (maximum), and *p*-value showing statistically significant differences in means between males and females.

Distance	Total				Male				Female				*p*-value
	n	Mean	SD (CV%)	95% CI [min, max]	n	Mean	SD (CV%)	95% CI [min, max]	n	Mean	SD (CV%)	95% CI [min, max]	
Right main bronchus (mm)	54	19.7	5.0 (25.2)	18.3-21.0 [9.6, 32.4]	28	21.4	5.3 (24.7)	19.4-23.5 [11.8, 32.4]	26	17.8	3.9 (21.9)	16.2-19.4 [9.6, 29.9]	0.006
Left main bronchus (mm)	55	44.2	5.3 (12.1)	42.7-45.6 [33.6, 55.3]	26	44.8	5.1 (11.4)	42.8-46.9 [33.6, 55.3]	29	43.6	5.5 (12.7)	41.5-45.7 [34.3, 54.1]	0.398

Supplemental Digital Content 1B: Correlation between parameters including height and airway parameters with their linear regression Pearson's correlation included the analyses between height and all airway parameters and between some unrelated pairs of airway parameters with their correlation coefficients (*r*) and statistical significance level (*p*-value) provided. Linear regression results (r^2 and regressed equation) were shown only in pairs with statistical significance (p-value < 0.05).

	**Pearson's Correlation Coefficient (*p*-value)**				**Linear Regression**	
	**Height**	**Subglottis**	**Trachea**	**Right primary bronchus**	**r^2**	**Equation**
**Right primary bronchus (mm)**	0.316 (0.020)	0.111 (0.422)	-0.133 (0.338)		0.100	0.202 (height in cm) - 12.812
**Left primary bronchus (mm)**	0.247 (0.069)	0 (0.999)	0.270 (0.046)	0.300 (0.051)	0.073	0.183 (trachea in mm) + 26.318

Supplemental Digital Content 2: Endotracheal tube characteristics (data presented in mm)

Size ID	Brand	OD	Mark-Tip distance		Mark-Cuff distance		Cuff-Tip distance	Murphy's eye
			Lower mark	Upper mark	Lower mark	Upper mark		
6.5	A	8.9	84.7 (0.4)	-	32.2 (0.7)	-	52.6	Yes
	B	8.7	79.6 (1.3)	-	24.6 (1.1)	-	55.0	Yes
	C	8.9	82.6 (0.6)	103.4 (0.5)	21.3 (0.7)	42.0 (1.0)	61.3	Yes
	D	8.8	77.0 (0.6)	86.8 (0.6)	19.2 (0.9)	29.1 (1.0)	57.7	Yes
	E	8.8	64.9 (0.3)	69.8 (0.3)	18.0 (0.2)	22.8 (0.3)	46.9	Yes
7.0	A	9.6	90.6 (0.7)	-	32.6 (1.9)	-	57.9	Yes
	B	9.3	85.5 (0.3)	-	24.8 (0.5)	-	60.7	Yes
	C	9.5	82.1 (1.2)	102.7 (0.7)	19.6 (0.5)	40.7 (1.0)	62.2	Yes
	D	9.6	85.8 (0.9)	95.6 (1.1)	22.7 (1.1)	32.3 (1.2)	63.2	Yes
	E	9.6	68.7 (0.3)	73.6 (0.4)	16.4 (0.4)	21.2 (0.6)	52.4	Yes
	F	9.3	78.8 (0.5)	88.8 (0.4)	20.3 (0.3)	30.0 (0.7)	58.6	Yes
	G	9.5	82.6 (0.4)	-	22.2 (0.8)	-	60.4	Yes
7.5	A	10.3	89.9 (1.2)	-	31.6 (2.5)	-	58.3	Yes
	B	10.0	89.4 (0.5)	-	23.1 (1.2)	-	66.4	Yes
	C	10.2	85.0 (0.4)	105.8 (0.6)	19.4 (1.0)	40.0 (0.7)	65.7	Yes
	D	10.2	88.7 (0.7)	98.7 (0.7)	22.5 (1.5)	32.4 (1.5)	66.3	Yes
	E	10.2	72.1 (0.4)	82.0 (0.4)	21.2 (0.7)	30.8 (0.7)	51.0	Yes
	F	10.0	81.2 (0.8)	91.1 (0.7)	20.7 (0.2)	30.3 (0.4)	60.6	Yes
	G	10.2	84.2 (0.1)	-	22.5 (0.8)	-	61.7	Yes
8.0	A	10.9	97.4 (0.8)	-	37.7 (3.6)	-	59.7	Yes
	B	10.7	90.6 (1.1)	-	23.7 (0.7)	-	66.9	Yes
	C	10.8	89.7 (0.3)	111.1 (0.4)	21.2 (0.7)	42.1 (1.1)	68.7	Yes
	D	10.9	92.0 (0.9)	101.8 (0.9)	23.9 (0.7)	33.8 (0.9)	68.0	Yes
	E	10.9	74.8 (0.4)	84.6 (0.4)	21.8 (1.5)	31.5 (1.5)	53.0	Yes
	F	10.7	83.9 (1.1)	93.8 (1.0)	20.6 (0.4)	30.3 (0.8)	63.4	Yes
8.5	A	11.6	98.1 (0.4)	-	36.8 (1.7)	-	61.3	Yes
	B	11.3	94.9 (0.9)	-	25.8 (3.8)	-	69.1	Yes
	C	11.4	92.7 (1.3)	114.0 (1.1)	21.1 (0.9)	41.6 (0.9)	72.0	Yes
	D	11.5	94.9 (0.9)	104.6 (0.7)	26.4 (1.9)	36.1 (1.7)	68.5	Yes
	E	11.5	73.7 (0.5)	83.7 (0.4)	21.3 (0.3)	30.7 (0.5)	52.7	Yes

Remark: Data presented as mean (SD), measurement in mm; ID, Inner Diameter; OD, Outer Diameter; Mark-Cuff = vocal cord marking to the upper edge of cuff; Mark-Tip = vocal cord marking to the tip of the endotracheal tube; Cuff-Tip = the upper edge of cuff to the tip of the endotracheal tube

A: Portex® (Ref 100/199/065-085, Smiths Medical International Ltd., Hythe, Kent, UK);

B: Ruschelit® (Ref 100382, Teleflex Medical Sdn. Bhd., Kamunting, Perak, Malaysia);

C: Shiley™ TaperGuard (Ref 18765-18785, Covidien llc, Mansfield, MA, USA);

D: Curity® (Ref 9465E-9485E, Covidien llc, Mansfield, MA, USA);

E: Unomedical™ (Ref UM61110065-85, Well Lead Medical Co., Ltd., Guangzhou, P.R.China);

F: Fornia™ (Ref QG-P2-7.0 to 8.0, Royal Fornia Medical Equipment, Co., Ltd., Guangdong, P.R. China);

G: MICROCUFF® (Ref 35214-5, Avanos Medical Inc., Alpharetta, GA, USA).

Supplemental Digital Content 3: Expected mark-cuff (Z_1) and expected mark-tip (Z_2) distances calculated based on height. (Created with BioRender.com)

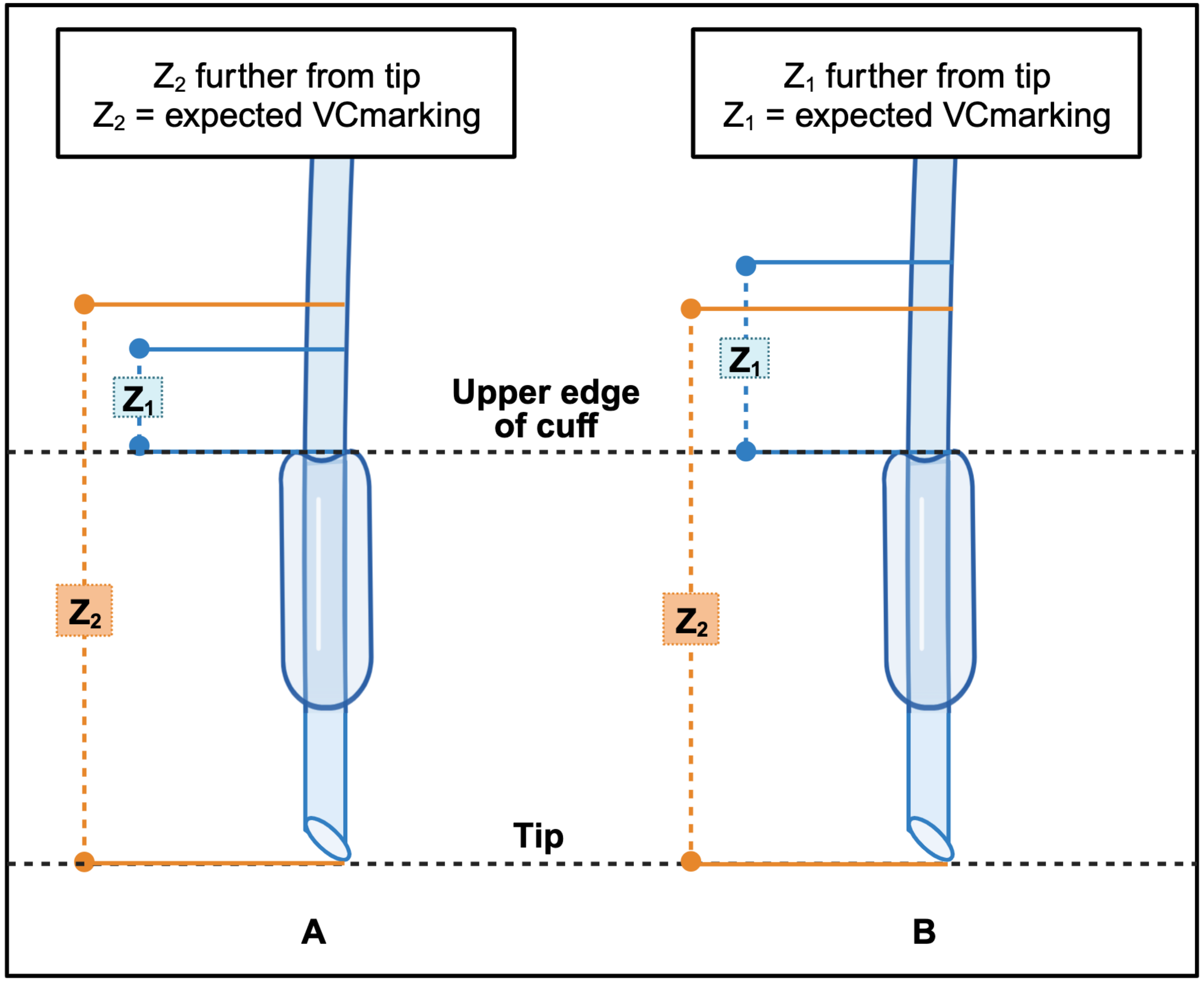

Supplemental Digital Content 4: Cuff position and tip position from endotracheal tube placement simulation in different scenarios.

Supplemental Digital Content 4A: Cuff and tip position when the endotracheal tube was placed using vocal cord marking in males (21 simulations, N=40).

Sim No.	Brand	Cuff position (Fig 3A)								Tip position (Fig 4A)							
		Cuff in subglottis				CO-Cuff < 2 cm				Endobronchial intubation				Tip-Carina < 2 cm			
		N	%	95% CI lower	95% CI upper	N	%	95% CI lower	95% CI upper	N	%	95% CI lower	95% CI upper	N	%	95% CI lower	95% CI upper
	**Male ID 7.5**																
1	A	1	2.5	0.4	12.9	39	97.5	87.1	99.6	0	0	0.0	8.8	2	5	1.4	16.5
2	B	33	82.5	68.1	91.3	7	17.5	8.7	31.9	0	0	0.0	8.8	1	2.5	0.4	12.9
3	C-Lower	39	97.5	87.1	99.6	1	2.5	0.4	12.9	0	0	0.0	8.8	1	2.5	0.4	12.9
4	C-Upper	0	0	0.0	8.8	38	95	83.5	98.6	1	2.5	0.4	12.9	22	55	39.8	69.3
5	D-Lower	35	87.5	73.9	94.5	5	12.5	5.5	26.1	0	0	0.0	8.8	1	2.5	0.4	12.9
6	D-Upper	1	2.5	0.4	12.9	39	97.5	87.1	99.6	1	2.5	0.4	12.9	7	17.5	8.7	31.9
7	E-Lower	36	90	76.9	96.0	4	10	4.0	23.1	0	0	0.0	8.8	0	0	0.0	8.8
8	E-Upper	2	5	1.4	16.5	38	95	83.5	98.6	0	0	0.0	8.8	1	2.5	0.4	12.9
9	F-Lower	38	95	83.5	98.6	2	5	1.4	16.5	0	0	0.0	8.8	1	2.5	0.4	12.9
10	F-Upper	3	7.5	2.6	19.9	37	92.5	80.1	97.4	0	0	0.0	8.8	2	5	1.4	16.5
11	G	35	87.5	73.9	94.5	5	12.5	5.5	26.1	0	0	0.0	8.8	1	2.5	0.4	12.9

Supplemental Digital Content 4A (continued)

Sim No.	Brand	Cuff position (Fig 3A)								Tip position (Fig 4A)							
		Cuff in subglottis				CO-Cuff < 2 cm				Endobronchial intubation				Tip-Carina < 2 cm			
		N	%	95% CI lower	95% CI upper	N	%	95% CI lower	95% CI upper	N	%	95% CI lower	95% CI upper	N	%	95% CI lower	95% CI upper
	**Male ID 8.0**																
12	A	0	0	0.0	8.8	40	100	91.2	100.0	0	0	0.0	8.8	5	12.5	5.5	26.1
13	B	31	77.5	62.5	87.7	9	22.5	12.3	37.5	0	0	0.0	8.8	2	5	1.4	16.5
14	C-Lower	36	90	76.9	96.0	4	10	4.0	23.1	0	0	0.0	8.8	2	5	1.4	16.5
15	C-Upper	0	0	0.0	8.8	35	87.5	73.9	94.5	2	5	1.4	16.5	28	70	54.6	81.9
16	D-Lower	29	72.5	57.2	83.9	11	27.5	16.1	42.8	0	0	0.0	8.8	3	7.5	2.6	19.9
17	D-Upper	0	0	0.0	8.8	40	100	91.2	100.0	1	2.5	0.4	12.9	13	32.5	20.1	48.0
18	E-Lower	35	87.5	73.9	94.5	5	12.5	5.5	26.1	0	0	0.0	8.8	0	0	0.0	8.8
19	E-Upper	1	2.5	0.4	12.9	39	97.5	87.1	99.6	0	0	0.0	8.8	1	2.5	0.4	12.9
20	F-Lower	38	95	83.5	98.6	2	5	1.4	16.5	0	0	0.0	8.8	1	2.5	0.4	12.9
21	F-Upper	3	7.5	2.6	19.9	37	92.5	80.1	97.4	0	0	0.0	8.8	3	7.5	2.6	19.9

Supplemental Digital Content 4B: Cuff and tip position when the endotracheal tube was placed using vocal cord marking in females (22 simulations, N=40).

Sim No.	Brand	Cuff position (Fig 3A)								Tip position (Fig 4A)							
		Cuff in subglottis				CO-Cuff < 2 cm				Endobronchial intubation				Tip-Carina < 2 cm			
		N	%	95% CI lower	95% CI upper	N	%	95% CI lower	95% CI upper	N	%	95% CI lower	95% CI upper	N	%	95% CI lower	95% CI upper
	**Female ID 7.0**																
1	A	0	0	0.0	8.8	40	100	91.2	100.0	0	0	0.0	8.8	4	10	4.0	23.1
2	B	10	25	14.2	40.2	30	75	59.8	85.8	0	0	0.0	8.8	2	5	1.4	16.5
3	C-Lower	33	82.5	68.1	91.3	7	17.5	8.7	31.9	0	0	0.0	8.8	0	0	0.0	8.8
4	C-Upper	0	0	0.0	8.8	28	70	54.6	81.9	1	2.5	0.4	12.9	24	60	44.6	73.7
5	D-Lower	20	50	35.2	64.8	20	50	35.2	64.8	0	0	0.0	8.8	2	5	1.4	16.5
6	D-Upper	0	0	0.0	8.8	40	100	91.2	100.0	0	0	0.0	8.8	15	37.5	24.2	53.0
7	E-Lower	39	97.5	87.1	99.6	1	2.5	0.4	12.9	0	0	0.0	8.8	0	0	0.0	8.8
8	E-Upper	27	67.5	52.0	79.9	13	32.5	20.1	48.0	0	0	0.0	8.8	0	0	0.0	8.8
9	F-Lower	29	72.5	57.2	83.9	11	27.5	16.1	42.8	0	0	0.0	8.8	0	0	0.0	8.8
10	F-Upper	1	2.5	0.4	12.9	39	97.5	87.1	99.6	0	0	0.0	8.8	4	10	4.0	23.1
11	G	23	57.5	42.2	71.5	17	42.5	28.5	57.8	0	0	0.0	8.8	1	2.5	0.4	12.9

Supplemental Digital Content 4B (continued)

Sim No.	Brand	Cuff position (Fig 3A)								Tip position (Fig 4A)							
		Cuff in subglottis				CO-Cuff < 2 cm				Endobronchial intubation				Tip-Carina < 2 cm			
		N	%	95% CI lower	95% CI upper	N	%	95% CI lower	95% CI upper	N	%	95% CI lower	95% CI upper	N	%	95% CI lower	95% CI upper
	**Female ID 7.5**																
12	A	0	0	0.0	8.8	40	100	91.2	100.0	0	0	0.0	8.8	4	10	4.0	23.1
13	B	20	50	35.2	64.8	20	50	35.2	64.8	0	0	0.0	8.8	4	10	4.0	23.1
14	C-Lower	34	85	70.9	92.9	6	15	7.1	29.1	0	0	0.0	8.8	2	5	1.4	16.5
15	C-Upper	0	0	0.0	8.8	31	77.5	62.5	87.7	2	5	1.4	16.5	29	72.5	57.2	83.9
16	D-Lower	22	55	39.8	69.3	18	45	30.7	60.2	0	0	0.0	8.8	4	10	4.0	23.1
17	D-Upper	0	0	0.0	8.8	40	100	91.2	100.0	0	0	0.0	8.8	22	55	39.8	69.3
18	E-Lower	27	67.5	52.0	79.9	13	32.5	20.1	48.0	0	0	0.0	8.8	0	0	0.0	8.8
19	E-Upper	0	0	0.0	8.8	40	100	91.2	100.0	0	0	0.0	8.8	0	0	0.0	8.8
20	F-Lower	28	70	54.6	81.9	12	30	18.1	45.4	0	0	0.0	8.8	0	0	0.0	8.8
21	F-Upper	0	0	0.0	8.8	40	100	91.2	100.0	0	0	0.0	8.8	5	12.5	5.5	26.1
22	G	22	55	39.8	69.3	18	45	30.7	60.2	0	0	0.0	8.8	1	2.5	0.4	12.9

Supplemental Digital Content 4C: Cuff position when the tip of the endotracheal tube was placed at mid-trachea in males (13 simulations, N=40).

Sim No.	Brand	Tip at mid-trachea (Fig 3B)							
		Cuff in subglottis				CO-Cuff < 2 cm			
		N	%	95% CI lower	95% CI upper	N	%	95% CI lower	95% CI upper
	**Male ID 7.5**								
1	A	39	97.5	87.1	99.6	1	2.5	0.4	12.9
2	B	40	100	91.2	100.0	0	0	0.0	8.8
3	C	40	100	91.2	100.0	0	0	0.0	8.8
4	D	40	100	91.2	100.0	0	0	0.0	8.8
5	E	26	65	49.5	77.9	14	35	22.1	50.5
6	F	40	100	91.2	100.0	0	0	0.0	8.8
7	G	40	100	91.2	100.0	0	0	0.0	8.8
	**Male ID 8.0**								
8	A	40	100	91.2	100.0	0	0	0.0	8.8
9	B	40	100	91.2	100.0	0	0	0.0	8.8
10	C	40	100	91.2	100.0	0	0	0.0	8.8
11	D	40	100	91.2	100.0	0	0	0.0	8.8
12	E	32	80	65.2	89.5	8	20	10.5	34.8
13	F	40	100	91.2	100.0	0	0	0.0	8.8

Supplemental Digital Content 4D: Cuff position when the tip of endotracheal tube was placed at mid-trachea in females (14 simulations, N=40).

Sim No.	Brand	Tip at mid-trachea (Fig 3B)							
		Cuff in subglottis				CO-Cuff < 2 cm			
		N	%	95% CI lower	95% CI upper	N	%	95% CI lower	95% CI upper
	**Female ID 7.0**								
1	A	39	97.5	87.1	99.6	1	2.5	0.4	12.9
2	B	39	97.5	87.1	99.6	1	2.5	0.4	12.9
3	C	40	100	91.2	100.0	0	0	0.0	8.8
4	D	40	100	91.2	100.0	0	0	0.0	8.8
5	E	33	82.5	68.1	91.3	7	17.5	8.7	31.9
6	F	39	97.5	87.1	99.6	1	2.5	0.4	12.9
7	G	39	97.5	87.1	99.6	1	2.5	0.4	12.9
	**Female ID 7.5**								
8	A	39	97.5	87.1	99.6	1	2.5	0.4	12.9
9	B	40	100	91.2	100.0	0	0	0.0	8.8
10	C	40	100	91.2	100.0	0	0	0.0	8.8
11	D	40	100	91.2	100.0	0	0	0.0	8.8
12	E	28	70	54.6	81.9	12	30	18.1	45.4
13	F	39	97.5	87.1	99.6	1	2.5	0.4	12.9
14	G	40	100	91.2	100.0	0	0	0.0	8.8

Supplemental Digital Content 4E: Tip position when the endotracheal tube was placed using expected Mark-Cuff (Z_1) calculation to avoid cuff in the subglottis in males (13 simulations, N=40), Z_1 = 0.173*(height in cm) + 4.453.

Sim No.	Brand	Z_1 by calculation (Fig 4B)							
		Endobronchial intubation				Tip-Carina < 2 cm			
		N	%	95% CI lower	95% CI upper	N	%	95% CI lower	95% CI upper
	**Male ID 7.5**								
1	A	0	0	0.0	8.8	3	7.5	2.6	19.9
2	B	1	2.5	0.4	12.9	8	20	10.5	34.8
3	C	0	0	0.0	8.8	7	17.5	8.7	31.9
4	D	1	2.5	0.4	12.9	8	20	10.5	34.8
5	E	0	0	0.0	8.8	1	2.5	0.4	12.9
6	F	0	0	0.0	8.8	3	7.5	2.6	19.9
7	G	0	0	0.0	8.8	3	7.5	2.6	19.9
	**Male ID 8.0**								
8	A	0	0	0.0	8.8	3	7.5	2.6	19.9
9	B	1	2.5	0.4	12.9	9	22.5	12.3	37.5
10	C	1	2.5	0.4	12.9	12	30	18.1	45.4
11	D	1	2.5	0.4	12.9	11	27.5	16.1	42.8
12	E	0	0	0.0	8.8	1	2.5	0.4	12.9
13	F	0	0	0.0	8.8	5	12.5	5.5	26.1

Supplemental Digital Content 4F: Tip position when the endotracheal tube was placed using expected Mark-Cuff (Z_1) calculation to avoid cuff in the subglottis in females (14 simulations, N=40), $Z_1 = 0.173*(\text{height in cm}) + 4.453$.

Sim No.	Brand	Z_1 by calculation (Fig 4B)							
		Endobronchial intubation				Tip-Carina < 2 cm			
		N	%	95% CI lower	95% CI upper	N	%	95% CI lower	95% CI upper
	**Female ID 7.0**								
1	A	0	0	0.0	8.8	4	10	4.0	23.1
2	B	0	0	0.0	8.8	5	12.5	5.5	26.1
3	C	0	0	0.0	8.8	10	25	14.2	40.2
4	D	0	0	0.0	8.8	13	32.5	20.1	48.0
5	E	0	0	0.0	8.8	2	5	1.4	16.5
6	F	0	0	0.0	8.8	4	10	4.0	23.1
7	G	0	0	0.0	8.8	5	12.5	5.5	26.1
	**Female ID 7.5**								
8	A	0	0	0.0	8.8	4	10	4.0	23.1
9	B	0	0	0.0	8.8	19	47.5	32.9	62.5
10	C	0	0	0.0	8.8	17	42.5	28.5	57.8
11	D	0	0	0.0	8.8	19	47.5	32.9	62.5
12	E	0	0	0.0	8.8	0	0	0.0	8.8
13	F	0	0	0.0	8.8	5	12.5	5.5	26.1
14	G	0	0	0.0	8.8	8	20	10.5	34.8

Remark:

A: Portex® (Ref 100/199/065-085, Smiths Medical International Ltd., Hythe, Kent, UK);

B: Ruschelit® (Ref 100382, Teleflex Medical Sdn. Bhd., Kamunting, Perak, Malaysia);

C: Shiley™ TaperGuard (Ref 18765-18785, Covidien llc, Mansfield, MA, USA);

D: Curity® (Ref 9465E-9485E, Covidien llc, Mansfield, MA, USA);

E: Unomedical™ (Ref UM61110065-85, Well Lead Medical Co., Ltd., Guangzhou, P.R.China);

F: Fornia™ (Ref QG-P2-7.0 to 8.0, Royal Fornia Medical Equipment, Co., Ltd., Guangdong, P.R. China);

G: MICROCUFF® (Ref 35214-5, Avanos Medical Inc., Alpharetta, GA, USA).
